# Single-Center Experience With Endovascular Treatment for Splenic Artery Aneurysms in Long-Term Follow-Up: A Retrospective Study

**DOI:** 10.3389/fcvm.2021.793053

**Published:** 2022-01-28

**Authors:** Yanyan Cao, Songlin Song, Tao Ouyang, Chuansheng Zheng

**Affiliations:** ^1^Department of Radiology, Union Hospital, Tongji Medical College, Huazhong University of Science and Technology, Wuhan, China; ^2^Hubei Province Key Laboratory of Molecular Imaging, Wuhan, China

**Keywords:** splenic, artery aneurysm, endovascular treatment, individual-tailed, long-term follow-up

## Abstract

**Objectives:**

To reveal a single-center experience with endovascular treatment for splenic artery aneurysm (SAA) and analyze the safety and efficacy of the operation in the long-term follow-up.

**Materials and Methods:**

A total of 49 patients with SAAs (21 men, 28 women; mean age, 52.4 ± 11.5 years) were enrolled in this study from July 2010 to December 2020. Baseline and characteristics of SAAs were collected. Parent artery coil embolization or combined with sac coil embolization of SAAs, graft-stent implantation, or bare-stent-assisted coil embolization were performed for the treatment of SAAs. Adverse events and follow-up data were recorded.

**Results:**

The average diameter of SAAs was 3.3 ± 2.5 cm (range, 1.0–13.6 cm). An individual-tailed modality was conducted for three patients. A 100% technical success rate was achieved. No re-intervention procedure was performed in all patients. No major treatment-related adverse events were observed, and no expansion or rupture of SAAs occurred in the average follow-up period of 57.9 ± 27.3 months (19–125 months).

**Conclusion:**

Endovascular treatment of SAA, including the individual-tailed therapy for three cases, is safe, effective, and minimally invasive with high technical success rates and satisfactory outcomes during the long-term follow-up period.

## Introduction

Splenic artery aneurysm (SAA) is a rare vascular disorder with an incident rate of 0.1–2.0%, which accounts for 50–60% in all visceral aneurysms (1–3). Rupture is the most dangerous complication of SAA, with a mortality of up to 10–25% (4). Surgery used to be the standard treatment method for SAA, but endovascular management become the first option in recent years for the advantages of minimally invasive, highly successful rates of operation, and fewer complications (5–8). However, the long-term outcomes of patients with SAA who are treated with endovascular treatment (EVT) remain unclear. This study aims to investigate the long-term efficiency and safety of different interventional modalities among 49 patients with SAA in our center during the maximum follow-up period of 125 months. Besides, clinical experience of EVT in some cases was particularly discussed.

## Materials and Methods

### Patient Selection and Ethics

The study continued from July 2010 to December 2020 and consecutively included data from patients with SAA diagnosed by CT angiography or ultrasonography and who received EVT in our center. Inclusion criteria were as follows: (1) diameter of a true SAA larger than 2 cm; (2) asymptomatic but the diameter increases more than 0.5 cm annually; (3) asymptomatic but with pregnancy intention; (4) symptomatic regardless of the diameter; and (5) pseudoaneurysm. Exclusion criteria were as follows: (1) with other diseases affecting the prognosis, such as the dysfunction of vital organs or cancer; (2) receiving treatment in other centers; and (3) missing data. The study was conducted according to the Declaration of Helsinki. Informed consent was waived for the retrospective analysis of this study. The information of all participants was maintained with confidentiality.

### Intervention

Surgical interventions were performed by operators with at least 5 years of experience in this field. All the patients underwent splenic artery angiography through a 5 French catheter (Cook, Bloomington, Indiana, USA) by puncturing the femoral artery. After confirming the location, size, shape, and the parent artery of SAAs, patients were intervened with one of the following procedures: (i) simple coil embolization of the aneurysm sac; (ii) coil embolization of the parent artery, including embolization of the proximal and distal parent artery, namely isolation embolization; (iii) sac coil embolization combined with isolation embolization; (iv) stent-graft implantation; and (v) bare-stent-assisted coil embolization. Complications of EVT, including abdominal pain, nausea, and fever were assessed and treated. All patients receiving coil embolization were given broad-spectrum antibiotics after the procedure, and those intervened by graft implantation received aspirin or clopidogrel for 3 months.

### Follow-Up

Patients were followed up at 3 and 12 months after the operation and annually thereafter by CT angiography. Complications, reinterventions, and mortality during the follow-up period were recorded and analyzed. All patients were followed up until death, loss to follow-up, or by the end of follow-up.

## Results

### Baseline and SAAs Characteristics

There were 3 patients excluded for receiving treatments in other centers and 6 for suffering SAAs smaller than 1 cm. A total of 49 patients with SAA received EVT in our center (Table 1), including 21 men and 28 women with a mean age of 52.4 ± 11.5 years. There were 61 aneurysms in 49 patients with SAA, of which 18, 12, 29, and 2 SAAs were located at the proximal, middle, distal splenic artery, and within spleen parenchyma, respectively. The average diameter of SAAs was 3.3 ± 2.5 cm (range, 1.0–13.6 cm). There were 41 patients with true SAAs, of which 26 had pregnancy history, 11 had atherosclerosis, and 6 had portal hypertension, some patients among them developed several complications. Besides, there were 8 patients with pseudoaneurysms, among which 2 had hypertension, 2 acute pancreatitides, 1 chronic pancreatitis, 1 subtotal gastrectomy, and 1 abdominal trauma history. There were 4 and 5 patients with true and pseudo-SAAs had symptoms (e.g., abdominal pain and bloating) before treatment, respectively.

**Table 1:** Baseline characteristics of patients and splenic artery aneurysms (SAAs).

**Demographics**	**No**.
**Gender**	
Male	21 (42.9)
Female	28 (57.1)
**Age** (years; mean ± SD, range)	52.4 ± 11.5 (27–70)
**Symptoms**	
Yes	23 (46.9)
No	26 (53.1)
**Comorbidities**	
Pregnancy history	28 (57.1)
Atherosclerosis	13 (26.5)
Hypertension	7 (14.3)
Portal hypertension	6 (12.2)
**Pancreatitis**	
Acute	2 (4.1)
Chronic	1 (2.0)
Pancreatic lesion	2 (4.1)
Trauma or gastrectomy history	2 (4.1)
**Type of SAAs**	
True	41 (83.7)
Pseudo-	8 (16.3)
**Location of SAAs**	
Proximal	18 (36.7)
Middle	12 (24.5)
Distal	29 (59.2)
Spleen parenchyma	2 (4.1)
Total	61
Diameter of SAAs (cm; mean ± SD, range)	3.3 ± 2.5 (1.0–13.6)

### EVT Data

**Figure 1:**
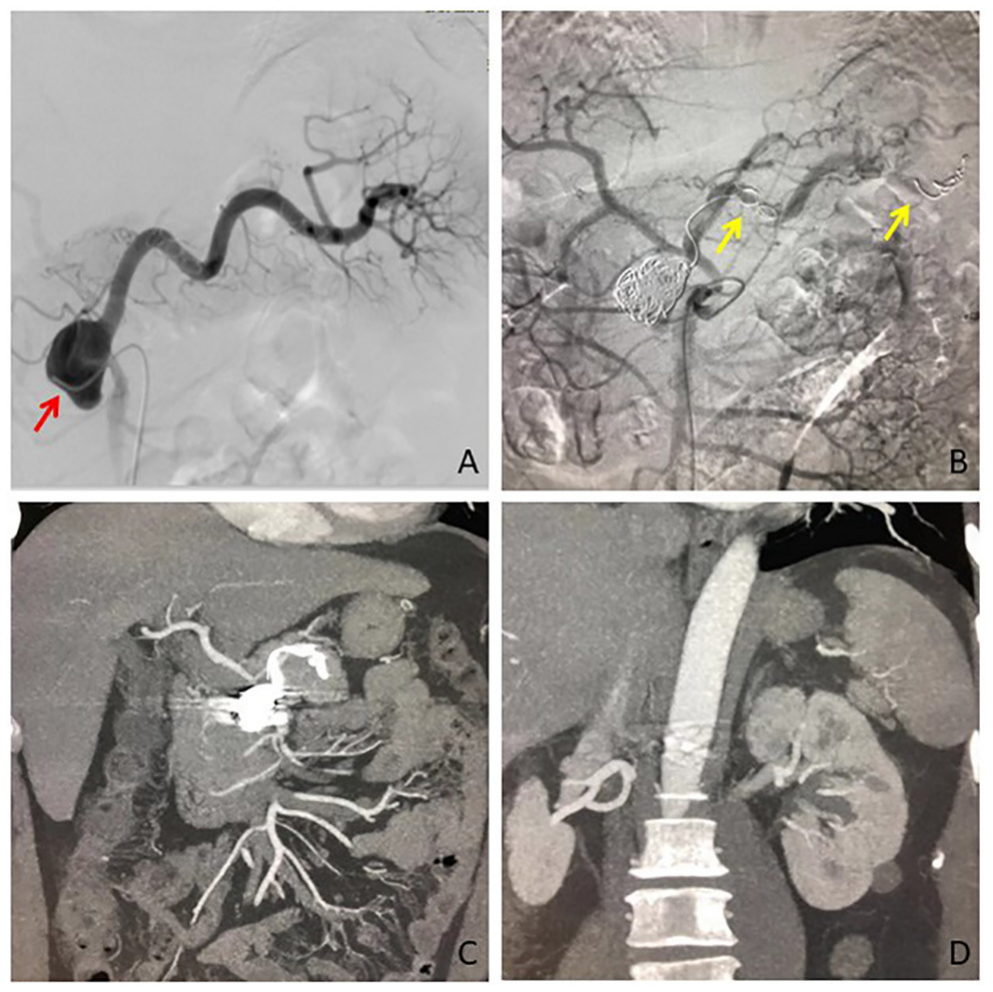
The SAA was diagnosed in a patient with the symptom of abdominal discomfort. **(A)** Digital subtraction angiography confirmed the location of SAA at the initiation of the splenic artery, which arose from the superior mesenteric artery (red arrow). **(B)** The distal side of the SAA parent artery, aneurysm sac, and the proximal side was embolized by coils (yellow arrow) in sequence. **(C)** CT angiography at 3 months showed completely aneurysmal thrombosis, and **(D)** No splenic infarction occurred. SAA, splenic artery aneurysm.

**Figure 2:**
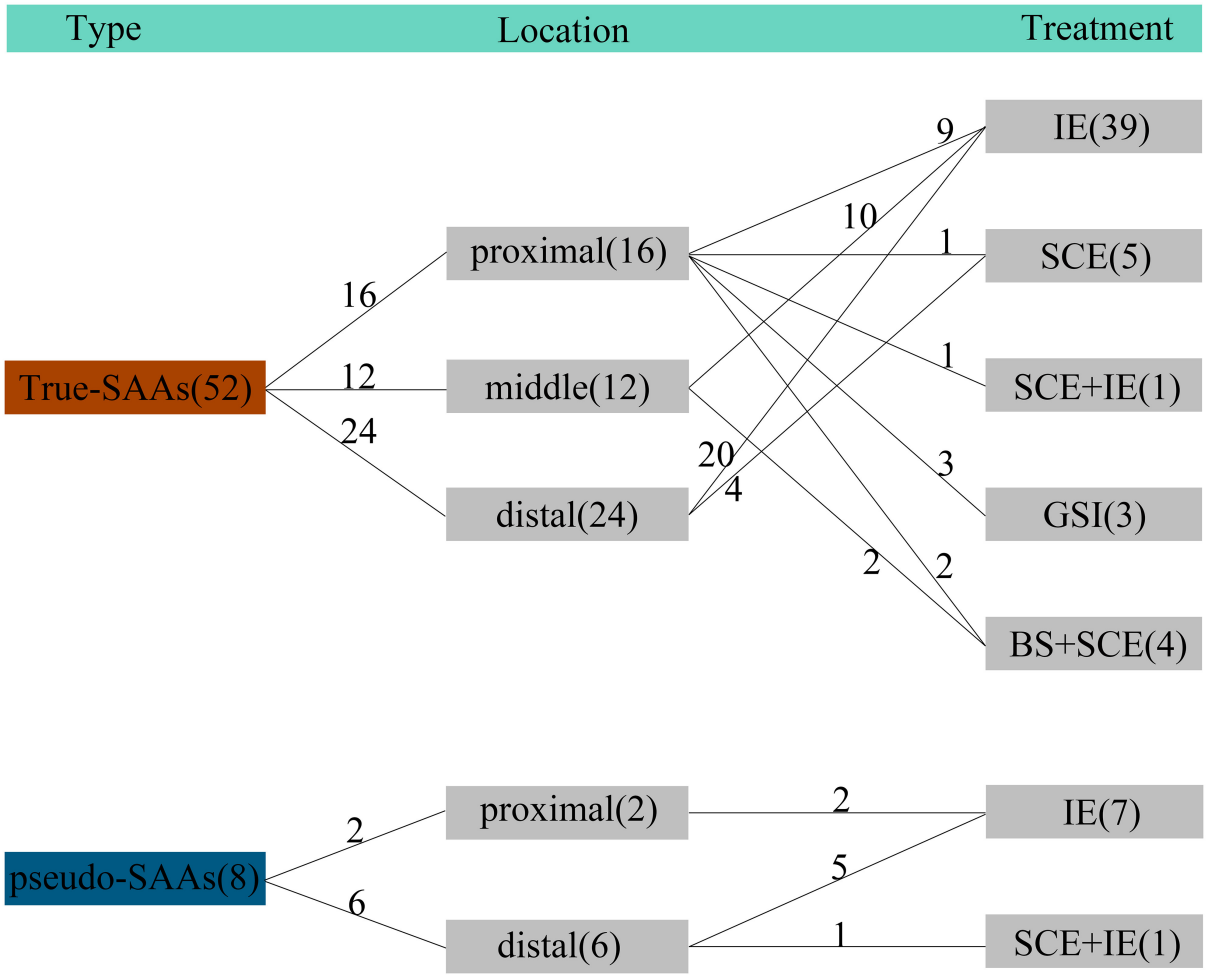
The characteristics of SAAs and treatment methods adopted in this study. There were 52 true SAAs included, and 16 SAAs among them located at the proximal parent artery, which received treatment of IE (*n* = 9), SCE (*n* = 1), SCE + IE (*n* = 1), GSI (*n* = 3), and BS + SCE (*n* = 2), respectively. For SAAs located at the middle parent (*n* = 12), they received IE (*n* = 10) and BS + SCE (*n* = 2), respectively. For SAAs located at the distal parent artery (*n* = 24), they received IE (*n* = 20) and SCE (*n* = 4), respectively. There were 8 pseudo-SAAs included and the 2 SAAs located at the proximal side both received IE treatment. The remained 6 pseudo-SAAs received IE (*n* = 5) and SCE + IE (*n* = 1) treatment, respectively. IE, isolation embolization; SCE, sac coil embolization; GSI, graft-stent implantation; BS, bare-stent.

All the patients received interventional therapy of EVT and the success rate was 100%. No treatment-related death occurred. There were 36, 2, and 4 patients who received isolation embolization, sac coil embolization combined with isolation embolization (Figure 1), and sac coil embolization, respectively. Besides, graft-stent implantation and bare-stent-assisted coil embolization were performed in 3 and 2 patients with SAA, respectively. All the data were summarized in Figure 2. The average length of stay was 6.4 ± 3.0 days. In particular, the stay was longer in SAA patients with comorbidities like pancreatitis, portal hypertension complicated with ascites, and cholecystitis than those without comorbidities [10.7 ± 6.4 days (13–22 days) vs. 5.5 ± 3.2 days (3–9 days)].

### Adverse Events

No major adverse events were observed according to the Society of Interventional Radiology classification system (9). Mild postembolization syndromes were observed in 15 patients (Table 2) but recovered a few days later spontaneously or after treatment. Twelve patients among them developed splenic infarction of varied areas (15%−35%), including 11 patients who intervened with isolation embolization and 1 with sac coil embolization combined with isolation embolization, respectively. No splenic abscess was observed. The discomfort was not observed in patients who received stent implantation. A transient elevation of serum amylase on the third day postoperatively was observed in 2 patients with pancreatitis, and they recovered to baseline 1 week later.

**Table 2:** Treatment and outcomes of 49 patients with SAAs.

	**Isolation embolization**	**Sac coil embolization + isolation embolization**	**Sac coil embolization**	**Graft-stent implantation**	**Bare-stent + sac coil implantation**	**Total**
Patients	36 (73.5%)	2 (4.1%)	4 (8.2%)	3 (6.1%)	4 (8.2%)	49 (100%)
Postembolization symptoms	14 (38.9%)	1 (50%)	0	0	0	15 (30.6%)
Splenic infarction	11 (30.6%)	1 (50%)	0	0	0	12 (24.5%)
Serum amylase elevation	2 (5.6%)	0	0	0	0	2 (4.1%)
Hospital stays	7.1 ± 3.2	5.5 ± 0.7	4.0 ± 0.8	3.7 ± 0.6	5.5 ± 0.6	6.4 ± 3.0

### Follow-Up

The average follow-up period was 57.9 ± 27.3 months (range, 19–125 months). No rupture was reported and no reintervention procedure was performed in all patients. All the aneurysms were thrombosed during the follow-up period, while the blood flow returned normal without embolization or stenosis after stent implantation.

## Discussion

Splenic artery aneurysms are categorized into true and pseudoaneurysms (10). Although the etiology of SAA is poorly understood, atherosclerosis, hypertension, portal hypertension, and pregnancy are generally considered risk factors (11–13). Besides, pancreatitis, trauma, and history of abdomen surgery are closely related to splenic artery pseudoaneurysm (12). Baseline characteristics of patients in the study were consistent with the above-mentioned risk factors. A consensus has been made about the treatment indications of SAAs (14, 15), such as the diameter of a true SAA is larger than 2 cm, symptomatic regardless of the diameter, and pseudoaneurysm. All the recruited patients with SAA were eligible to receive treatment based on the consensus. This study indicated that EVT was safe and efficacy in the long-term follow-up, which has not been reported previously. The transient elevation of serum amylase in 2 patients could be attributed to the embolization of common vessels which the spleen and pancreas shared.

The endovascular treatment method was adopted according to the presentation of angiography. The distal parent artery of some giant SAAs was small and tortuous, which increased the risk of introducing the guidewire to the distal site. Then a modified individual-tailed method was conducted in our center. That is, a 10% larger coil than the parent artery of SAA was introduced in the catheter after which was placed in the proximal side of the aneurysm. Normal saline was injected to push the coil forward, and the coil generally stopped at the distal parent artery at first. Then, repeated the procedure until the coils in the sac of SAA were immovable. The proximal side was embolized at last to block up the feeding artery of the aneurysm. There were 3 patients who received the modified individual-tailed method and achieved a similar thrombogenesis compared with other techniques. No aberrant adverse events or reintervention were reported in the follow-up period, indicating the safety and efficacy of this newly modified method, despite that has not been reported previously. It was also practical in SAA cases with several distal parent arteries owing to less invasive procedures and short surgical duration.

Besides, several conventional therapeutic procedures were used, each tailored by specific morphological configurations. For SAAs with a narrow neck or saccular shape on the middle and distal artery, simple coil embolization of aneurysm sac until no contrast medium retention is preferred, and keeping parent blood-flow fluent as much as possible. However, rupture or reperfusion of the aneurysms may be caused by an excessive packing or less densely embolization, respectively. Based on clinical experience in our center, a complete embolization of the aneurysmal sac is performed by securing it using a large coil, which was then densely packed using a same or smaller size coil to make a “nest,” thus reducing the potential shift and keeping fluent bloodstream in the parent artery. In addition, platinum coils and solely controlled detachment coils could plug the aneurysms more efficiently, showing a more superb performance than that of steel coils for the acceptable flexibility, less displacement, and reduced incidence of rupture. In this study, 4 patients receiving sac coil embolization achieved a satisfactory outcome. However, simple sac coil embolization poses a high risk of rupture in some aneurysm cases, such as the pseudoaneurysm (16). A combination of parent artery embolization and packing coils less densely in the sac would be a better choice.

On the other hand, for SAAs with wide-neck (>4 mm) or spindle-shaped appearance (17), one of the following procedures could be conducted according to the location of SAAs. (1) If SAAs are located at the major splenic artery, graft-stent implantation with the appropriate size is better to block the opening of SAA and keep fluent blood flow at the same time. However, a 7 French guiding catheter to introduce the graft-stent, a closure device (e.g., angioseal) to stitch the puncture point in the femoral artery, and an anticoagulant were essential for the procedure. There were 3 patients who adopted graft-stent implantation with a straight parent artery without splenic infarction in long-term follow-up. (2) If SAAs are located at the branch of the major splenic artery, or where the graft-stent is too hard to reach, then the procedure of bare-stent-assisted coil embolization could be conducted due to its flexibility. There are two modalities to achieve EVT. First, releasing the bare-stent after coil embolization, that is, the microcatheter was inserted into the aneurysm lumen first, then a bare-stent was released across the neck, and the microcatheter was withdrawn after the aneurysm lumen was embolized densely by coils. There were 4 patients who received this treatment, and no thrombus in the stent or splenic infarction was observed during the follow-up period. Second, releasing the bare-stent is followed by coil embolization, which is sometimes difficult to introduce the microcatheter to sac through the mesh of bare-stent technically. (3) If the parent artery is located at the artery branch of segment or bifurcation of splenic hilum, the embolization of parent artery by polyvinyl alcohol or coils is preferred, or performing isolation embolization to exclude reperfusion. In addition, the pipeline embolization device may be another choice for wide-neck SAAs, which have been proven its efficacy in intracranial aneurysms (18), but the study in SAAs is poor.

There are several limitations to this study. First, this was a retrospective, single-center study about EVT in the management of SAA with small sample size. Second, the sample size of patients with SAA treated with stent placement was limited, and as a result, clinical experience was scant. Third, we did not have the experience of dealing with the tissue adhesive during the procedure of EVT, although it has been previously reported in some studies mentioned.

In conclusion, EVT for SAA was safe, effective, and minimally invasive as evidenced by a high technical success rate and satisfactory long-term follow-up outcomes. In addition, the individual-tailed management strategy is beneficial to the prognosis of SAA, despite which needs more cases to further demonstrate its efficacy and safety.

## Data Availability Statement

The raw data supporting the conclusions of this article will be made available by the authors, without undue reservation.

## Ethics Statement

Ethical review and approval was not required for the study on human participants in accordance with the local legislation and institutional requirements. Written informed consent for participation was not required for this study in accordance with the national legislation and the institutional requirements.

## Author Contributions

All authors listed have made a substantial, direct, and intellectual contribution to the work and approved it for publication.

## Conflict of Interest

The authors declare that the research was conducted in the absence of any commercial or financial relationships that could be construed as a potential conflict of interest.

## Publisher's Note

All claims expressed in this article are solely those of the authors and do not necessarily represent those of their affiliated organizations, or those of the publisher, the editors and the reviewers. Any product that may be evaluated in this article, or claim that may be made by its manufacturer, is not guaranteed or endorsed by the publisher.
